# Cutaneous Metastasis: A Rare Presentation of Pancreatic Adenocarcinoma

**DOI:** 10.7759/cureus.69767

**Published:** 2024-09-19

**Authors:** Prabhat Nichkaode, Divyansh Dwivedi

**Affiliations:** 1 Department of General Surgery, Dr. D. Y. Patil Medical College, Hospital and Research Centre, Dr. D. Y. Patil Vidyapeeth (Deemed to be University), Pune, IND

**Keywords:** adenocarcinoma, gastroenterology, general surgery, pathophysiology, route, tumor markers

## Abstract

Pancreatic adenocarcinomas are highly malignant tumors. The liver, peritoneum, and, very rarely, the skin are the sites of pancreatic metastases. Cutaneous metastasis is rare, with scalp metastasis being seldomly found. We report the case of an elderly woman who initially presented with ulcerated scalp swellings. The immunohistochemistry analysis of the swollen scalp confirmed the presence of a far-metastasized adenocarcinoma. Imaging of the abdomen performed due to increasing cholestatic jaundice revealed adenocarcinoma located at the head of the pancreas. Cholestatic jaundice was treated by trans-biliary percutaneous drainage. She is currently on a chemotherapy regimen. The patient had a follow-up appointment one month ago for the fourth cycle of gemcitabine and paclitaxel and will continue to follow up until eight cycles of chemotherapy sessions. Cutaneous metastases resulting from pancreatic cancer are a rare occurrence and may occasionally present as the only visible symptom; therefore, they should be included in the differential diagnosis of skin lesions.

## Introduction

Pancreatic adenocarcinoma is one of the highly malignant tumors with higher rates of metastasis. The pancreas is a long, thin organ that is situated in the retroperitoneum. That is why, at presentation, the disease is usually locally advanced or metastasized. It is well known that pancreatic cancer spreads quickly, usually to the liver and peritoneum, then to the lungs, bones, and brain [1,2]. Sporadic cutaneous metastases are uncommon, but when present, they are known as Sister Mary Joseph's nodule (SMJN), seen near the umbilicus [3]. There are very few documented occurrences of non-umbilical cutaneous metastases [4,5]. Here, we present a unique instance of scalp swellings diagnosed to be metastasis from pancreatic adenocarcinoma. The patient also had numerous lung and liver metastases but had no other symptoms on presentation. To the best of our knowledge, this is the first case of pancreatic adenocarcinoma with scalp metastasis.

## Case presentation

**Figure 1:**
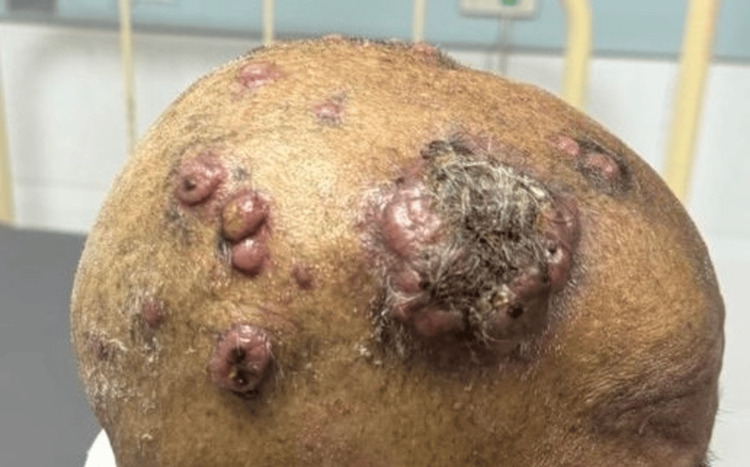
Multiple swellings over the scalp

A 59-year-old woman presented to our hospital with multiple ulcerated lesions all over her scalp (Figure 1). She also complained of jaundice and pruritus for the past 10 days. There is no history of abdominal pain, abdominal distension, fever, or prodromal symptoms. She gives a history of weight loss of over 5 kg in six months. History revealed the recent onset of type 2 diabetes mellitus for the past six months. On local scalp examination, multiple swellings were seen, the largest measuring 4 x 4 cm, and the rest were approximately 1 x 2 cm and 2 x 2 cm. The nodules were ulcerated with minimal discharge. The examination of the abdomen revealed hepatomegaly, measuring 2 cm below the costal margin. No other lymph nodes were palpable. The per rectal examination was normal.

The routine blood chemistry revealed obstructive jaundice, while the renal function test and electrolytes were within the normal range (Table 1).

**Table 1:** Laboratory investigations CEA: carcinoembryonic antigen; CA19-9: carbohydrate antigen 19-9

Investigations	Result	Reference Value
Hemoglobin	12.7 g/dL	11.6-15 g/dL
Total leukocyte count	10,600 cells/μL	4000-10000 cells/μL
Platelet count	2,20,000 cells/μL	150000-410000/μL
Total bilirubin	16.04 mg/dL	0.22-1.2 mg/dL
Direct bilirubin	14 mg/dL	Up to 0.5 mg/dL
Aspartate transaminase	380 U/L	8-43 U/L
Alanine transaminase	202 U/L	7-45 U/L
Alkaline phosphatase	1404 U/L	35-104 U/L
Gamma-glutamyl transferase	463 U/L	5-36 U/L
Amylase	25 U/L	25-115 U/L
Lipase	24 U/L	8-78 U/L
CEA	7.33 ng/mL	Less than 3 ng/mL
CA19-9	32.86 U/L	Less than 37 U/mL

**Figure 2:**
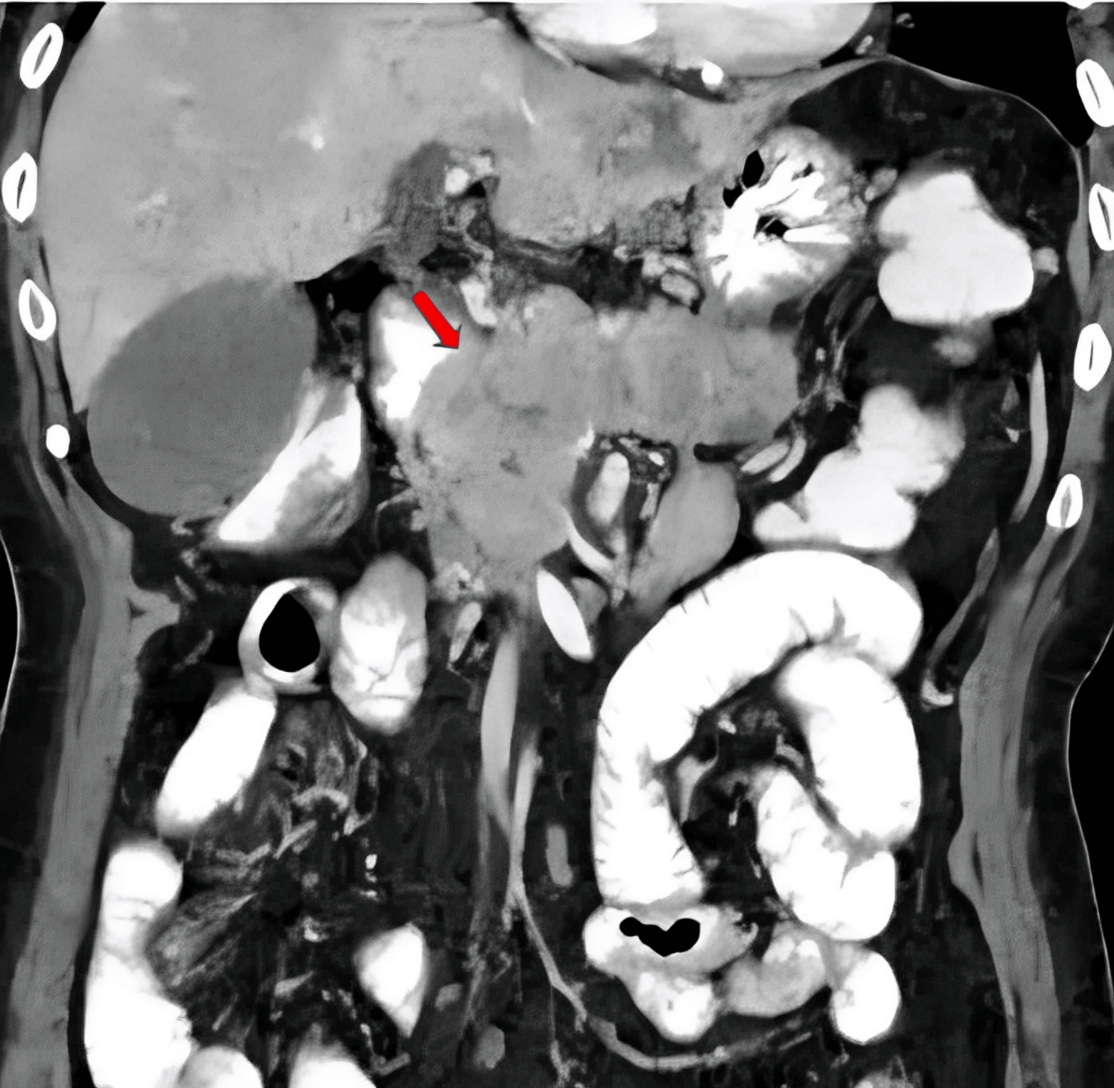
Contrast-enhanced computed tomography of the abdomen showing a mass located in the head of the pancreas (red arrow)

Imaging of the thorax and abdomen was performed due to suspected cancer. The contrast-enhanced computed tomography scan of the abdomen showed a tumor in the pancreatic head with obstructive biliary dilatation. Metastases were seen in the retroperitoneal lymph nodes, bilateral adrenal glands, and at multiple sites in the lung and liver (Figure 2).

**Figure 3:**
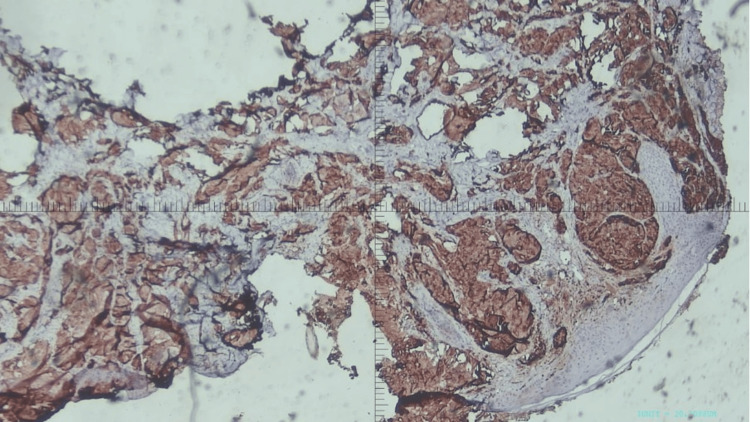
Histopathology showing adenocarcinoma of the pancreas Immunohistochemistry showed a positive reaction to CK 19, CK 7, CK 20, CDX-2, and CEA. These markers revealed metastatic adenocarcinoma of pancreaticobiliary origin. CK: creatine kinase; CDX: cyclin-dependent kinases; CEA: carcinoembryonic antigen

Meanwhile, the skin biopsy immunohistochemical staining showed a positive reaction to creatine kinase (CK) 19, CK 7, CK 20, cyclin-dependent kinases (CDX-2), and carcinoembryonic antigen (CEA). These markers revealed metastatic adenocarcinoma of pancreaticobiliary origin (Figure 3).

Subsequently, endoscopic ultrasound-guided fine-needle aspiration cytology was conducted. An endoscopic ultrasound showed an oval and heterogeneous mass measuring 32 × 28 mm located in the head of the pancreas. It caused compression of the lower common bile duct and dilation of the intra-hepatic biliary radicals.

The pancreatic biopsy's histological results, which revealed the growth of tiny, atypical gland ducts, suggest that pancreatic ductal adenocarcinoma with clear cytoplasm was the main cancer. Immunohistochemical staining of the biopsy showed a positive reaction to CK 19, CK 7, CK 20, CDX-2, and CEA, suggestive of pancreatic metastatic carcinoma. The morphology of the occipital ulcer tissue closely resembled that of the main tumor, allowing us to identify the tumor as a metastatic cutaneous tumor that originated from pancreatic cancer.

Due to developing cholangitis and the non-feasibility of endoscopic retrograde pancreatography, the patient underwent percutaneous trans-biliary drainage to drain the left biliary system with a micropuncture set. Liver function test improved significantly post-drainage. After achieving bilirubin levels of 3 mg/dL, the patient was started on chemotherapy. Advanced pancreatic cancer with distant metastases was diagnosed and treated with a combination of gemcitabine and paclitaxel; chemotherapy is currently effectively progressing as there have been no unusual side effects or deterioration in the patient's overall health since the diagnosis.

The patient last followed up one month ago for the fourth cycle of gemcitabine and paclitaxel and will continue to follow up until eight cycles of chemotherapy sessions. However, there was no decrease in the size of the scalp metastasis.

## Discussion

High global mortality rates are observed in people diagnosed with pancreatic adenocarcinoma with metastases. Metastasis typically affects the brain, liver, peritoneum, lymph nodes in the region, and lungs. Atypical locations of pancreatic cancer metastases are the muscle, skin, heart, pleura, and stomach [6].

Pancreatic carcinoma-related cutaneous metastases are particularly uncommon. Lookingbill et al. [7] found that just two cases (0.48%) out of 420 patients with cutaneous metastases had pancreatic origins. At autopsy, cutaneous metastases were found in 9/119 patients (7.6%) with pancreatic cancer, according to Cubilla and Fitzgerald [8]. According to Miyahara et al., skin lesions were seen in the umbilicus in 16 out of 22 patients (72.7%) where pancreatic cancer had metastasized cutaneously [9]. Our case is distinct because, as opposed to the umbilicus, the scalp had an uncommon cutaneous metastasis of pancreatic cancer as its initial clinical presentation.

It is quite challenging to identify the primary cause of occult tumor-related cutaneous metastases. Nonetheless, the doctor might be able to accurately identify the main tumor with immunohistochemistry. The most sensitive tumor marker for pancreatic adenocarcinoma is CA19-9, which was first discovered in colorectal cancer. This antigen is also present in non-cancerous illnesses and other malignancies.

Ridwelski et al. suggested that monoclonal antibodies against cytokeratin may be more accurate and specific when identifying disseminated tumor cells in lymph nodes than CA19-9 [10]. This is particularly relevant as there exists a relationship between the level of CA19-9 and the false positivity or false negatives for CA19-9. There are, in fact, numerous varieties of CK that are exclusive to different organs. The endocrine islets, duct cells, and exocrine acinar cells in the pancreas are responsible for producing CK 8 and CK 18. Typically, only the ductal cells have CK 17 and CK 19. The cytokeratin immunophenotype of pancreaticobiliary ductal adenocarcinomas is largely similar to that of normal pancreatic ducts; this includes being positive for CK 7, 8, 18, and 1916.

Approximately 50% and 90%, respectively, of pancreaticobiliary adenocarcinomas stain diffusely with CK 19 and CK 7 antibodies [11]. According to recent research by Duval et al., the majority of extrahepatic and pancreatic carcinomas tested positive for CK 7 but negative for CK 20 [12]. As a result, it is thought that the information on the immunohistochemistry expression of CKs is helpful in the identification of metastatic carcinomas.

There are several theories regarding cutaneous metastasis; however, no particular mechanism has been identified. Some of these theories include the chemotaxis hypothesis, direct invasion, lymphatic or hematogenous dispersion, and the soil and seed hypothesis.

## Conclusions

In conclusion, pancreatic cancer rarely presents as cutaneous metastases. However, this possibility should be considered in the differential diagnosis, particularly when the malignant skin lesion is of unknown origin. Clinicians need to be aware that cutaneous lesions with metastases may be the first indication of pancreatic cancer. Additionally, the diagnosis of metastatic pancreatic adenocarcinoma may benefit from the use of CK 7 and 19 immunohistochemistry staining.
